# SINS/BDS tightly coupled integrated navigation algorithm for hypersonic vehicle

**DOI:** 10.1038/s41598-022-10063-9

**Published:** 2022-04-12

**Authors:** Kai Chen, Sen-sen Pei, Cheng-zhi Zeng, Gang Ding

**Affiliations:** 1 School of Astronautics, Northwestern Polytechnical University, Xi’an, 710072 China; 2 China Airborne Missile Academy, Luoyang, 471009 China

**Keywords:** Aerospace engineering, Engineering

## Abstract

A tightly coupled integrated navigation system (TCINS) for hypersonic vehicles is proposed when the satellite signals are disturbed. Firstly, the architecture of the integrated navigation system for the hypersonic vehicle is introduced. This system applies fiber SINS, BeiDou satellite receiver (BDS) and System On a Parogrammable Chip (SOPC) missile-born computer. Subsequently, the SINS mechanization for hypersonic vehicle is presented. The J2 model is employed for the normal gravity of the near space. An algorithm for updating the attitude, velocity and position is designed. State equations and measurement equations of SINS/BDS tightly coupled integrated navigation for hypersonic vehicle are given, and a scheme of validity for satellite data is designed. Finally, the SINS/BDS tightly coupled vehicle field tests and hardware-in-the-loop (HWIL) simulation tests are carried out. The vehicle field test and HWIL simulation results show that the heading angle error of tightly coupled integrated navigation is within 0.2°, the pitch and roll angle errors are within 0.05°, the maximum velocity error is 0.3 m/s, and the maximum position error is 10 m.

## Introduction

Near space vehicles have advantages that vehicles such as traditional aircrafts and satellites do not have, and have great development potential, especially in terms of intelligence collection and long-range precision strike. Near space vehicles can be divided into low-dynamic and high-dynamic vehicles^1^. Hypersonic vehicle is the representative of the rapid development of high-dynamic vehicle in recent years^2^, which adopts aerodynamic configuration design with high lift-drag ratio and propulsion technology such as hypersonic gliding, rocket engine or scramjet to achieve hypersonic flight^3^.

The navigation system is one of important parts in hypersonic vehicle, and is used to measure the position, velocity, and attitude of the vehicle. The strapdown inertial navigation system (SINS) has the advantages of full navigation information, high autonomy and high update rate. The GPS has global, all weather, constant and real-time navigation, positioning and timing functions^4^. Due to the inherent complementary advantages of SINS and Global Navigation Satellite System (GNSS), the navigation schemes of hypersonic vehicles in various countries around the world are all mainly based on SINS and GNSS^5^. X-43A hypersonic vehicles use Honeywell’s mature H-764 series INS/GPS system^6,7^, which is a self-contained, all-attitude, tightly coupled navigation system, and it can provide the position accuracy of less than 10 m and the speed accuracy less than 0.05 m/s in the case of integrated navigation. HTV-2 hypersonic vehicles apply a tightly coupled GPS/IMU integrated navigation system with an accuracy of about 3 m^8^. X-51A hypersonic vehicle is equipped with inertial measurement unit (IMU) and GPS receiver^9^. The navigation system of German SHEFEX-2 hypersonic vehicles integrates IMU, GPS receiver, and star sensor (STR), and uses IMU/GPS for integrated navigation^10^, and the star sensor of SHEFEX-2 aircraft starts to operate after the end of the boost phase, which effectively reduces the attitude error caused by the motion of the boost phase. Russian Avangard uses SINS/GLONASS/SAR integrated navigation scheme^3^, among them, GLONASS is used to update and correct INS, SAR is applied in the terminal stage of the trajectory. Hypersonic vehicle navigation systems in Japan and India also use SINS/GNSS integrated navigation systems.

The Chinese BeiDou Navigation Satellite System (BDS)^11^ has global and all-daytime navigation, and positioning and timing capabilities, and unique short message communication services. BDS has been extensively applied in marine fisheries, transportation, meteorological forecasting, hydrological monitoring, geographic information of surveying and mapping, and communication unify-time^12^. However, there are fewer application cases of BDS in hypersonic flight. In this paper, a SINS algorithm and a tightly coupled integrated navigation algorithm are designed for the SINS/BDS integrated navigation system for hypersonic vehicle, the processing strategy of the navigation system is given when the BeiDou satellite signal is disturbed, and vehicle field tests and hardware-in-the-loop simulation tests are also performed.

The contribution of this paper is organized as follows. Section “SINS/BDS integrated navigation system hardware” introduces the hardware of the SINS/BDS integrated navigation system. Section “SINS/BDS tightly coupled integrated navigation algorithm” describes the tightly coupled integrated navigation algorithm of SINS/BDS. Section “Test on SINS/BDS tightly coupled integrated navigation” carries out the vehicle field tests and hardware-in-the-loop (HWIL) simulation tests of the tight-coupled of SINS/BDS, and the simulation results are analyzed.

## SINS/BDS integrated navigation system hardware

### Overall structure of SINS/BDS integrated navigation system

**Figure 1:**
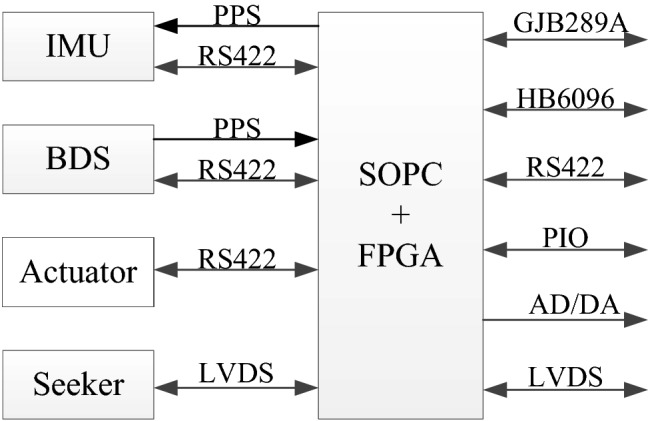
Composition of flight control components.

The SINS/BDS integrated navigation system is an important component of the flight control system in hypersonic vehicle. The flight control system includes a control system, a guidance system, and a navigation system. Flight control system hardware includes IMU, BeiDou satellite receiver, actuator, seeker, information processing unit, peripheral interface, etc. (Fig. 1).

### Information processing unit

The SOPC technology is applied for the integrated design for the information processing unit. The SOPC chip integrates an Advanced RISC Machines (ARM) processor, a million-gate programmable logic array, and an IP core with multiple bus communication functions to complete the information exchange between the system and the external interface. The software runs on the real-time operating system of the information processing unit, and runs integrated navigation algorithms, control algorithms, and guidance algorithms, and flight timing and process management.

### Inertial measurement unit

**Figure 2:**
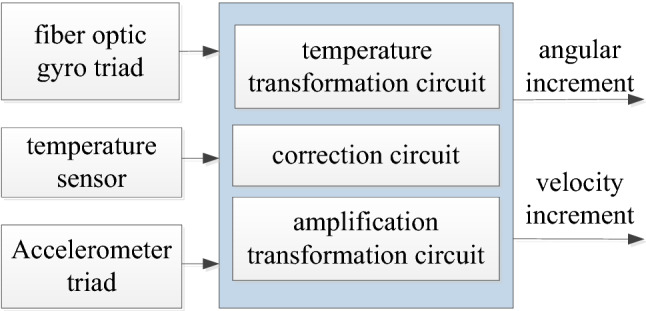
Functional principle diagram of the inertial measurement unit.

The inertial measurement unit (IMU) includes a three-axis fiber optic gyro cluster, a three-axis accelerometer cluster, an inertial measurement electronic cluster, and an inertial measurement secondary power supply (Fig. 2). The inertial measurement electronic cluster includes amplifier circuit, temperature measurement circuit, and correction circuit.

The IMU measures the three-axis acceleration and angular velocity of a hypersonic vehicle, and has the characteristics of high dynamics, large range, high accuracy, and miniaturization, where the fiber optic gyro bias is 3°/h(1σ) with 100 ppm(1σ) scale factor, and 0.5°/h(1σ) white noise, and the gyro data output period of 2 ms; and the accelerometer drift is 1 × 10^–3^ g(1σ) with the scale factor of 100 ppm(1σ), the white noise of 0.5 × 10^–4^ g(1σ), and the output period of 6 ms.

### BDS satellite receiver

**Figure 3:**
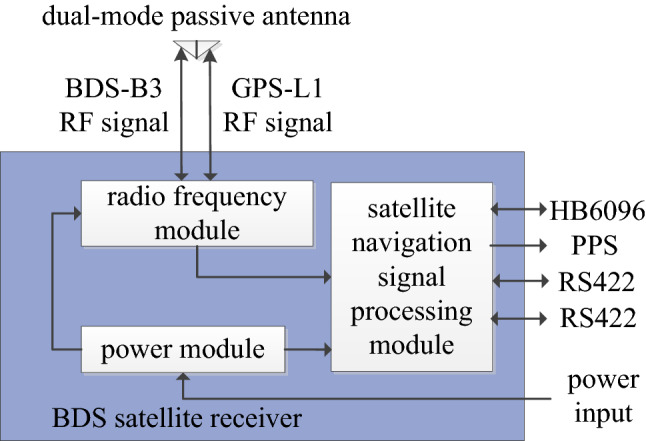
Diagram of the BDS satellite receiver.

The BDS satellite receiver uses the “radio frequency + ADC + FPGA + DSP” framework for the integrated design. FPGA is used for acquisition and track of BDS satellite navigation signals, and DSP is used for satellite navigation positioning and logic processing (Fig. 3). The receiver provides real-time velocity, position, and time information, as well as ephemeris, pseudo-range (PR), and pseudo-range rate (PRR) information for the hypersonic vehicle. At the same time, the positioning information is transmitted to the telemetry component for recording the flight trajectory of the hypersonic vehicle. The positioning accuracy of the satellite receiver is 10 m (1σ), the velocity accuracy is 0.3 m/s (1σ), and the output period is 200 ms.

## SINS/BDS tightly coupled integrated navigation algorithm

### Definition of coordinate systems

In this study, the following coordinate systems are used.

1. Coordinate system of the earth-centered inertial frame (ECI, *i* frame). The origin is the center of the earth, $x_{i}$- and $y_{i}$-axes are in the equatorial plane of the earth, $x_{i}$ points to towards the vernal equinox, and $z_{i}$ is the earth rotation axis through the conventional terrestrial pole.
2. Coordinate system of the earth-centered earth-fixed frame (ECEF, *e* frame). The origin is the center of the earth, $x_{e}$- and $y_{e}$-axes are in the equatorial plane of the earth, $x_{e}$ points to the prime meridian, and $z_{e}$ is the earth rotation axis.
3. Coordinate system of the body frame (*b* frame). The coordinate origin is the barycenter of the vehicle, the x-axis points to the head, the y-axis is in the main symmetry plane, and the up direction is positive. The body frame is the front-up-right coordinate system.
4. Coordinate system of the local-level frame (LL frame). The coordinate origin is the barycenter of the vehicle, the x-axis points to the east, the y-axis points to the north, the z-axis is perpendicular to the earth plane where the carrier is located and points to the sky.

### Overall frame of the TCINS algorithm

**Figure 4:**
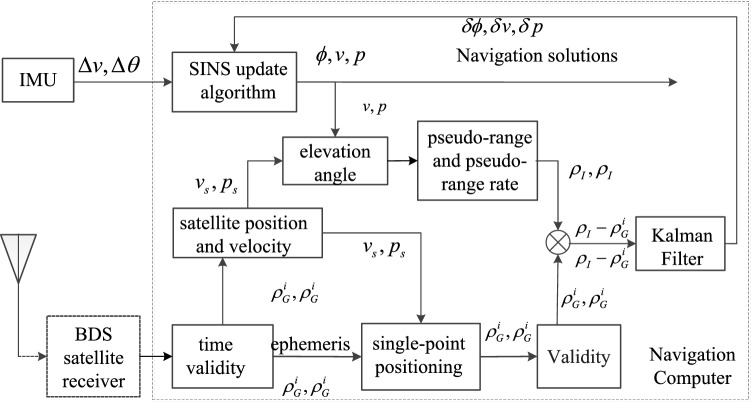
The data flow schematic diagram of the TCINS algorithm.

The data flow diagram of the SINS/BDS TCINS algorithm is shown in Fig. 4.

The implementation steps of the SINS/BDS TCINS algorithm are as follows.

1. Periodically carry out numerical update for SINS algorithm according to the angular and velocity increments output by the IMU, and output the attitude $\phi$, velocity $v$, and position $p$.
2. When receiving the data of the BDS satellite receiver, determine the receiving time and accuracy factor. If the data is valid, continue to step (3), otherwise return to step (1).
3. Perform incremental determination on the PR observations $\tilde{\rho }_{G}^{i}$ and PRR observations $\dot{\tilde{\rho }}_{G}^{i}$ of BDS receiver. If the data is valid, continue to step (4), otherwise return to step (1).
4. Use the ephemeris data, $\tilde{\rho }_{G}^{i}$, and $\dot{\tilde{\rho }}_{G}^{i}$ to calculate the position $p_{s}$ and velocity $v_{s}$ of the BDS receiver. Then calculate the satellite clock difference, ionospheric error, and tropospheric error based on the ephemeris data. Subsequently, perform single-point positioning to obtain the corrected $\rho_{G}^{i}$ and $\dot{\rho }_{G}^{i}$.
5. Use $v$, $p$, and ephemeris to calculate the satellite elevation angle. Select the appropriate satellite according to the satellite elevation angle, and calculate $\rho_{I}$ and $\dot{\rho }_{I}$ of SINS.
6. The difference between $\rho_{I}$ and $\dot{\rho }_{I}$ as well as the difference between $\rho_{G}^{i}$ and $\dot{\rho }_{G}^{i}$ are determined. If the determination is valid, they are combined through Kalman filtering, the error estimates $\delta \phi$, $\delta v$, and $\delta p$ are obtained, calculate SINS to output attitude $\phi$, velocity $v$, and position $p$, and perform correction. Otherwise return to step (1).

### Mechanization of hypersonic vehicle SINS algorithm

The mechanization of strapdown inertial navigation is to convert the output information of IMU into position, speed and attitude information through numerical operation. The local-level (LL) frame is choiced as the reference frame for hypersonic vehicle. The strapdown inertial navigation equation is shown in Eq. (1)^13^.

1 $$\left\{ {\begin{array}{*{20}l} {\dot{\varvec{q}}_{b}^{n} = \frac{1}{2}{\varvec{q}}_{b}^{n} \otimes \varvec{\omega}_{nb}^{b} } \hfill \\ {\dot{\varvec{v}}^{n} = {\varvec{R}}_{b}^{n} {\varvec{f}}^{b} - (2{\varvec{\omega}}_{ie}^{n} + {\varvec{\omega}}_{en}^{n} ) \times {\varvec{v}}^{n} + {\varvec{g}}^{n} } \hfill \\ {\dot{\varvec{p}}^{n} = {\varvec{C}}^{p} {\varvec{v}}^{n} } \hfill \\ \end{array} } \right.$$

where $${\varvec{q}}_{b}^{n}$$ is the attitude quaternion of the vehicle in the local-level frame; $${\varvec{\omega}}_{nb}^{b}$$ is the angular velocity; $${\varvec{v}}^{n}$$ is the velocity vector in LL frame; $${\varvec{R}}_{b}^{n}$$ is the Direction Cosine Matrix (DCM) of the body frame to the LL frame, $${\varvec{f}}_{{}}^{b}$$ is the specific force; $${\varvec{g}}^{n}$$ is the gravity vector of LL frame; $${\varvec{p}}^{n} = \left[ {\begin{array}{*{20}c} L & \lambda & h \\ \end{array} } \right]^{{\text{T}}}$$ is location, $${\varvec{C}}^{p} = \left[ {\begin{array}{*{20}c} 0 & {\frac{1}{{R_{M} + h}}} & 0 \\ {\frac{1}{{\left( {R_{N} + h} \right)\cos L}}} & 0 & 0 \\ 0 & 0 & 1 \\ \end{array} } \right],$$$$R_{M}$$ is the meridian radius and $$R_{M} = \frac{{a_{e} \left( {1 - e^{2} } \right)}}{{\left( {1 - e^{2} \sin^{2} L} \right)^{\frac{3}{2}} }}$$, $$R_{N}$$ is the normal radius and $$R_{N} = \frac{{a_{e} }}{{\left( {1 - e^{2} \sin^{2} L} \right)^{\frac{1}{2}} }}$$, $$a_{e}^{{}}$$ is the equatorial radius of the Earth, $$e$$ is the earth eccentricity.

Because the cruise altitude of the hypersonic vehicle is greater than the flight altitude of an aviation vehicle, this paper adopts the J_2_ gravity model in earth-centered earth-fixed (ECEF) frame^7,13^. The calculation method is shown in Eq. (2).

2 $$ {\varvec{g}}^{n} = {\varvec{R}}_{e}^{n} {\varvec{g}}^{e} $$

where ${\varvec{R}}_{e}^{n}$ is the DCM of the ECEF frame to the LL frame, as shown in Eq. (3). The vector calculation of ${\varvec{g}}^{e} = [g_{x}^{e} ,g_{y}^{e} ,g_{z}^{e} ]^{{\text{T}}}$ is shown in Eq. (4), and the calculation method is shown in Eq. (5).

3 $$ {\varvec{R}}_{e}^{n} = {\varvec{R}}_{z} \left( {90^{ \circ } } \right){\varvec{R}}_{y} \left( {90^{ \circ } - L} \right){\varvec{R}}_{z} \left( \lambda \right) $$

4 $$ {\varvec{g}}^{e} = {\varvec{G}}^{e} { + }{\varvec{\omega}}_{ie} \times {\varvec{\omega}}_{ie} \times {\varvec{r}}^{e} $$

5 $$ \left\{ {\begin{array}{*{20}l} {g_{x}^{e} = - \frac{{x_{e} \mu }}{{r^{3} }}\left[ {1 + \frac{{3J_{2} a_{e}^{2} }}{{2r^{2} }} - \frac{{15J_{2} a_{e}^{2} z_{e}^{2} }}{{2r^{4} }}} \right] + x_{e} \omega_{ie}^{2} } \hfill \\ {g_{y}^{e} = - \frac{{y_{e} \mu }}{{r^{3} }}\left[ {1 + \frac{{3J_{2} a_{e}^{2} }}{{2r^{2} }} - \frac{{15J_{2} a_{e}^{2} z_{e}^{2} }}{{2r^{4} }}} \right] + y_{e} \omega_{ie}^{2} } \hfill \\ {g_{z}^{e} = - \frac{{z_{e} \mu }}{{r^{3} }}\left[ {1 + \frac{{9J_{2} a_{e}^{2} }}{{2r^{2} }} - \frac{{15J_{2} a_{e}^{2} z_{e}^{2} }}{{2r^{4} }}} \right]} \hfill \\ \end{array} } \right. $$

In the Eq. (5), $\mu$ the Earth's gravitational coefficient, $J_{2}$ is the spherical harmonic coefficient of the Earth, $a_{e}^{{}}$ is the equatorial radius of the Earth, and $r = \sqrt {x_{e}^{2} { + }y_{e}^{2} { + }z_{e}^{2} }$. $x_{e} ,y_{e} ,z_{e}$ is calculated based on the latitude and longitude $\left( {L,\lambda ,h} \right)$ of the hypersonic vehicle, and calculated by the position ${\varvec{p}}^{e} { = }[x_{e} ,y_{e} ,z_{e} ]^{{\text{T}}}$ in the ECEF frame, as shown in Eq. (6).

6 $$ \left\{ {\begin{array}{*{20}l} {x_{e} = \left( {R_{N} + h} \right)\cos L\cos \lambda } \hfill \\ {y_{e} = \left( {R_{N} + h} \right)\cos L\sin \lambda } \hfill \\ {z_{e} = [R_{N} \left( {1 - e^{2} } \right) + h]\sin L} \hfill \\ \end{array} } \right. $$

### Strapdown inertial navigation numerical update algorithm

As mentioned above, the output periods of the gyro and the accelerometer are 2 ms and 6 ms, respectively. The strapdown inertial navigation algorithm update period $T$ is designed to be 6 ms. The gyroscope performs three equally interval samplings during the update cycle, and the angular increments are in order of ${\mathbf{\Delta \theta }}_{1}$, ${\mathbf{\Delta \theta }}_{3}$, and ${\mathbf{\Delta \theta }}_{3}$. The accelerometer samples once during the update cycle, and the velocity increment is ${{\varvec{\Delta}}}{\varvec{V}}$. The designed strapdown inertial navigation numerical update algorithm is as follows^14,15^.

#### Attitude update algorithm

According to the three samples of the gyroscope, the calculation method of rotation vector is

7 $${\varvec{\varPhi}}_{m} = \varvec{\Delta \theta }_{1} \varvec{ + \Delta \theta }_{2} \varvec{ + \Delta \theta }_{3} \varvec{ + }\frac{33}{{80}}\varvec{\Delta \theta }_{1} \times \varvec{\Delta \theta }_{3} + \frac{57}{{80}}\varvec{\Delta \theta }_{2} \times (\varvec{\Delta \theta }_{3} - \varvec{\Delta \theta }_{1} ) $$

Considering the rotation effect of the navigation coordinate system, the equivalent rotation vector ${\varvec{\eta}}_{m}$ from $t_{m - 1}$ to $t_{m}$ is

8 $$ {\varvec{\eta}}_{m} ={\varvec{\varPhi}}_{m} - {\varvec{R}}_{n(m - 1)}^{b} {\varvec{\omega}}_{in(m - 1)}^{n} T $$

The quaternion ${\varvec{q}}_{b(m)}^{b(m - 1)}$ corresponding to ${\varvec{\eta}}_{m}$ is

9 $$ {\varvec{q}}_{b(m)}^{b(m - 1)} = \cos \frac{{\eta_{m} }}{2} + \frac{{{\varvec{\eta}}_{m} }}{{\eta_{m} }}\sin \frac{{\eta_{m} }}{2} $$

Substitute the calculated ${\varvec{q}}_{b(m)}^{b(m - 1)}$ into Eq. (10) to complete the attitude update.

10 $$ {\varvec{q}}_{b(m)}^{n} = {\varvec{q}}_{b(m - 1)}^{n} {\varvec{q}}_{b(m)}^{b(m - 1)} $$

where ${\varvec{q}}_{b(m - 1)}^{n}$ is the attitude quaternion at $t_{m - 1}$, and ${\varvec{q}}_{b(m)}^{n}$ is the attitude quaternion at $t_{m}$.

#### Velocity update algorithm

From the specific force equation of Eq. (1), the velocity update algorithm can be obtained as follows.

11 $${\varvec{v}}_{m}^{n} = {\varvec{v}}_{m - 1}^{n} + \underbrace {{\int_{m - 1}^{m} {{\varvec{C}}_{b}^{n} {\varvec{f}}_{sf}^{b} } dt}}_{1} + \underbrace {{\int_{m - 1}^{m} {[{\varvec{g}}^{n} - (2\varvec{\omega}_{ie}^{n} + \varvec{\omega}_{en}^{n} ) \times {\varvec{v}}^{n} ]dt} }}_{2}$$

where the first term is caused by the velocity increment, which is $\varvec{\Delta v}_{sf(m)}^{n}$; and the second term is caused by the acceleration term, which is $\varvec{\Delta v}_{g/cor(m)}^{n}$.

The calculation method for the first term is

12 $$ \varvec{\Delta v}_{sf,m}^{n} = {\varvec{R}}_{n(m - 1)}^{n(m)} {\varvec{R}}_{b(m - 1)}^{n(m - 1)} \varvec{\Delta V} $$

where $${\varvec{R}}_{n(m - 1)}^{n(m)} \approx {\varvec{I}} - (\varvec{\varsigma }_{\frac{m}{2}} \times )$$, $$\varvec{\varsigma }_{\frac{m}{2}} = \frac{1}{2}\omega_{in(m - 1)}^{n} T$$.

The calculation method for the second term is

13 $$ \varvec{\Delta v}_{g/cor(m)}^{n} = [{\varvec{g}}_{m - 1}^{n} - (2{\varvec{\omega}}_{ie(m - 1)}^{n} + {\varvec{\omega}}_{en(m - 1)}^{n} ) \times {\varvec{v}}_{m - 1}^{n} ]T $$

Bring Eqs. (12) and (13) into Eq. (11) to complete the velocity update.

#### Position update algorithm

The position update is as shown below^7^.

14 $$ \left\{ {\begin{array}{*{20}l} {L_{m} = L_{m - 1} + \frac{{v_{N,m - 1}^{n} + v_{N,m}^{n} }}{{2(R_{M,m - 1} + h_{m - 1} )}}T} \hfill \\ {\lambda_{m} = \lambda_{m - 1} + \frac{{v_{E,m - 1}^{n} + v_{E,m}^{n} }}{{2(R_{N,m - 1} + h_{m - 1} )\cos L_{m - 1} }}T} \hfill \\ {h_{m} = h_{m - 1} + \frac{{v_{U,m - 1}^{n} + v_{U,m}^{n} }}{2}T} \hfill \\ \end{array} } \right. $$

### SINS/BDS tightly coupled filter

#### SINS/BDS tightly coupled state equation

SINS/BDS tightly coupled filter state vectors include attitude error ${\varvec{\phi}}$, velocity error $\delta {\varvec{v}}^{n}$, position error $\delta {\varvec{p}}$, gyroscope drift ${\varvec{\varepsilon}}^{b}$, and accelerometer drift $\varvec{\nabla }^{b}$, BDS pseudo-range error $\delta t_{u}$ and pseudo-range rate error $\delta t_{ru}$.

BDS main error comes from pseudo-range error $\delta t_{u}$ and pseudo-range rate error $\delta t_{ru}$, the pseudo-range error is equivalent to the clock error of BDS and pseudo-range rate error is equivalent to the clock frequency error, and it is expressed as:

15 $$ \left\{ {\begin{array}{*{20}l} {\delta \dot{t}_{u} = \delta t_{u} + \omega_{tu} } \hfill \\ {\delta \dot{t}_{ru} = - \beta_{{{\text{tru}}}} \delta t_{ru} + \omega_{tru} } \hfill \\ \end{array} } \right. $$

where is $\beta_{{{\text{tru}}}} = T_{ru}^{ - 1}$ first order Markov process correlation time, $\omega_{tu}$ and $\omega_{tru}$ are Gaussion white noise.

The state equation of the SINS/BDS filter is as follows. The state transition matrix ${\varvec{F}}(t)$ is described in references^16–18^.

16 $$ \dot{\varvec{X}}(t) = {\varvec{F}}(t){\varvec{X}}(t) + {\varvec{G}}(t){\varvec{W}}(t) $$

17 $$ \left[ {\begin{array}{*{20}c}    {\user2{\dot{\phi }}}  \\    {\delta \user2{\dot{v}}^{n} }  \\    {\delta \user2{\dot{p}}}  \\    {\user2{\dot{\varepsilon }}^{b} }  \\    {\user2{\dot{\nabla }}^{b} }  \\    {\delta \dot{t}_{u} }  \\    {\delta \dot{t}_{{ru}} }  \\   \end{array} } \right] = \left[ {\begin{array}{*{20}c}    { - (\user2{\omega }_{{in}}^{n}  \times )} & {\user2{F}_{1} } & {\user2{F}_{2} } & { - \user2{R}_{b}^{n} } & {{\mathbf{0}}_{{3 \times 3}} } & 0 & 0  \\    {(\user2{f}^{n}  \times )} & {\user2{F}_{3} } & {\user2{F}_{4} } & {{\mathbf{0}}_{{3 \times 3}} } & {\user2{R}_{b}^{n} } & 0 & 0  \\    {{\mathbf{0}}_{{3 \times 3}} } & {\user2{F}_{5} } & {\user2{F}_{6} } & {{\mathbf{0}}_{{3 \times 3}} } & {{\mathbf{0}}_{{3 \times 3}} } & 0 & 0  \\    {{\mathbf{0}}_{{3 \times 3}} } & {{\mathbf{0}}_{{3 \times 3}} } & {{\mathbf{0}}_{{3 \times 3}} } & {{\mathbf{0}}_{{3 \times 3}} } & {{\mathbf{0}}_{{3 \times 3}} } & 0 & 0  \\    {{\mathbf{0}}_{{3 \times 3}} } & {{\mathbf{0}}_{{3 \times 3}} } & {{\mathbf{0}}_{{3 \times 3}} } & {{\mathbf{0}}_{{3 \times 3}} } & {{\mathbf{0}}_{{3 \times 3}} } & 0 & 0  \\    0 & 0 & 0 & 0 & 0 & 1 & 0  \\    0 & 0 & 0 & 0 & 0 & 0 & { - \beta _{{tru}} }  \\   \end{array} } \right]\left[ {\begin{array}{*{20}c}    \user2{\phi }  \\    {\delta \user2{v}^{n} }  \\    {\delta \user2{p}}  \\    {\user2{\varepsilon }^{b} }  \\    {\user2{\nabla }^{b} }  \\    {\delta t_{u} }  \\    {\delta t_{{ru}} }  \\   \end{array} } \right] + \left[ {\begin{array}{*{20}c}    {\user2{I}_{{6 \times 6}} } & {{\mathbf{0}}_{{15 \times 2}} }  \\    {{\mathbf{0}}_{{11 \times 6}} } & {\user2{I}_{{2 \times 2}} }  \\   \end{array} } \right]\left[ {\begin{array}{*{20}c}    {\omega _{{gx}} }  \\    {\omega _{{gy}} }  \\    {\omega _{{gz}} }  \\    {\omega _{{ax}} }  \\    {\omega _{{ay}} }  \\    {\omega _{{az}} }  \\    {\omega _{{tu}} }  \\    {\omega _{{tru}} }  \\   \end{array} } \right] $$

where ${\varvec{F}}(t)$ is the state transition matrix, ${\varvec{X}}(t)$ is the state variable, ${\varvec{G}}(t)$ is the noise driving matrix, ${\varvec{W}}(t)$ is the process noise vector, ${\varvec{F}}_{1}$, ${\varvec{F}}_{2}$, ${\varvec{F}}_{3}$, ${\varvec{F}}_{4}$, ${\varvec{F}}_{5}$ and ${\varvec{F}}_{6}$ is the typical inertial parameter matrix, ${\varvec{f}}^{n}$ is the specific force.

#### SINS/BDS tightly coupled measurement equation

In SINS/BDS tightly coupled navigation system, SINS outgoing message and satellite ephemeris calculate SINS pseudo-range and pseudo-range rate, and BDS receives another set of pseudo-range and pseudo-range rate. The respective difference of pseudo range $\rho$ and pseudo-range rate $\dot{\rho }$ is taken as the measurement information of integrated filter. The measurement equation of SINS/BDS TCINS is^16,19^

18 $$ \begin{aligned}   \user2{Z}(t) &  = \left[ {\begin{array}{*{20}c}    {\user2{Z}_{\rho } (t)}  \\    {\user2{Z}_{{\dot{\rho }}} (t)}  \\   \end{array} } \right] = \left[ {\begin{array}{*{20}c}    {\user2{H}_{\rho } (t)}  \\    {\user2{H}_{{\dot{\rho }}} (t)}  \\   \end{array} } \right]\user2{X}(t) + \left[ {\begin{array}{*{20}c}    {\user2{V}_{\rho } (t)}  \\    {\user2{V}_{{\dot{\rho }}} (t)}  \\   \end{array} } \right] \\     &  = \left[ {\begin{array}{*{20}c}    {\begin{array}{*{20}c}    {\begin{array}{*{20}c}    {{\mathbf{0}}_{{m \times 6}} } & {\user2{ED}_{a} }  \\   \end{array} } & {{\mathbf{0}}_{{m \times 6}} } & {\user2{D}_{{tu}} } & {{\mathbf{0}}_{{m \times 1}} }  \\   \end{array} }  \\    {\begin{array}{*{20}c}    {\begin{array}{*{20}c}    {{\mathbf{0}}_{{m \times 3}} } & {\user2{ER}_{n}^{e} }  \\   \end{array} } & {{\mathbf{0}}_{{m \times 3}} } & {{\mathbf{0}}_{{m \times 7}} } & {\user2{D}_{{tu}} }  \\   \end{array} }  \\   \end{array} } \right]\user2{X}(t) + \left[ {\begin{array}{*{20}c}    {\user2{V}_{\rho } (t)}  \\    {\user2{V}_{{\dot{\rho }}} (t)}  \\   \end{array} } \right] \\  \end{aligned}  $$

where ${\varvec{Z}}(t)$ is the measurement vector, ${\varvec{Z}}_{\rho } (t) = \rho_{Ii} - \rho_{Bi}$, $\rho_{Ii}$ is the SINS pseudo-range, $\rho_{Bi}$ is the pseudo-range measured by BDS receiver, ${\varvec{Z}}_{{\dot{\rho }}} (t) = \dot{\rho }_{Ii} - \dot{\rho }_{Bi}$, $\dot{\rho }_{Ii}$ is the SINS pseudo-range rate, $\dot{\rho }_{Bi}$ is the pseudo-range rate of BDS receiver, ${\varvec{H}}(t)$ is the measurement matrix, ${\varvec{V}}(t)$ is the measurement noise, ${\varvec{E}}$, ${\varvec{D}}_{tu}$, ${\varvec{V}}_{\rho }$ and ${\varvec{V}}_{{\dot{\rho }}}$ are shown in Eq. (19), ${\varvec{D}}_{a}$ is shown in Eq. (20).

19 $$ {\varvec{E}} = \left[ {\begin{array}{*{20}c} {\begin{array}{*{20}c} {e_{1x} } \\ {e_{2x} } \\ \vdots \\ {e_{mx} } \\ \end{array} } & {\begin{array}{*{20}c} {e_{1y} } \\ {e_{2y} } \\ \vdots \\ {e_{my} } \\ \end{array} } & {\begin{array}{*{20}c} {e_{1z} } \\ {e_{2z} } \\ \vdots \\ {e_{mz} } \\ \end{array} } \\ \end{array} } \right]_{m \times 3} ,\quad {\varvec{D}}_{tu} = \left[ {\begin{array}{*{20}c} 1 \\ 1 \\ \vdots \\ 1 \\ \end{array} } \right]_{m \times 1} ,\quad {\varvec{V}}_{\rho } = \left[ {\begin{array}{*{20}c} {\upsilon_{\rho 1} } \\ {\upsilon_{\rho 2} } \\ \vdots \\ {\upsilon_{\rho m} } \\ \end{array} } \right]_{m \times 1} ,\quad {\varvec{V}}_{{\dot{\rho }}} = \left[ {\begin{array}{*{20}c} {\upsilon_{{\dot{\rho }1}} } \\ {\upsilon_{{\dot{\rho }2}} } \\ \vdots \\ {\upsilon_{{\dot{\rho }m}} } \\ \end{array} } \right]_{m \times 1} $$

20 $$ {\varvec{D}}_{a} = \left[ {\begin{array}{*{20}l} { - (R_{N} + h)\sin L\cos \lambda } \hfill &\quad { - (R_{N} + h)\cos L\sin \lambda } \hfill &\quad {\cos L\cos \lambda } \hfill \\ { - (R_{N} + h)\sin L\sin \lambda } \hfill &\quad {(R_{N} + h)\cos L\cos \lambda } \hfill &\quad {\cos L\sin \lambda } \hfill \\ {[R_{N} (1 - e^{2} ) + h]\cos L} \hfill &\quad 0 \hfill &\quad {\sin L} \hfill \\ \end{array} } \right] $$

where $\upsilon_{\rho i}$, $\upsilon_{{\dot{\rho }i}}$ respectively refers to pseudo-range and pseudo-range rate white noise output by Beidou receiver.

### Determination of satellite data validity

In order to ensure the accuracy of the SINS/BDS TCINS, effective satellite observations must be applied. However, the velocity of the near space hypersonic vehicle is over 5 Mach, which may result in abnormal values and observation anomaly in the satellite observations output by the BDS receiver, so as to affect the accuracy of the entire the TCINS. In this paper, the following four methods are applied to perform successive determination on the BDS receiver observations.

1. According to the receiving time
  The time of the BDS receiver output and that of the rising edge of the pulse per second (PPS) is not synchronized. The delay of the BDS receiver used in this paper is within 80 ms. If the time difference is less than the delay time, the package of BDS satellite observation data may be tightly coupled to the navigation; otherwise, the package is considered unusable^7^.
2. According to DOP and satellite elevation angle
  The satellite elevation angle is calculated based on the satellite position and the position calculated by the inertial navigation. Satellites with satellite elevation greater than 10° are valid satellites. If the dilution of precision (DOP) is less than 10 and the number of valid satellites is more than 2, the satellite observations are considered normal. Otherwise, the satellite observations are considered unusable.
3. According to pseudo-range and pseudo-range rate
  The PR and the PRR are calculated from SINS and satellite ephemeris at the current time, which are subtracted by the BDS PR observation and PRR observation at the same time. The validity of satellite data can be determined based on the differences and the thresholds.
4. According to the incremental of the measurement

The satellite observation data is continuously stored according to the satellite index. If the satellite index changes, the satellite observation data within 1 second cannot be used. If the satellite index does not change, calculate the increments of multi-packet PR and PRR that are continuously stored, and compare them with the increment threshold. The validity of satellite observation data can be determined through the incremental changes.

## Test on SINS/BDS tightly coupled integrated navigation

### SINS/BDS integrated navigation vehicle field test

**Figure 5:**
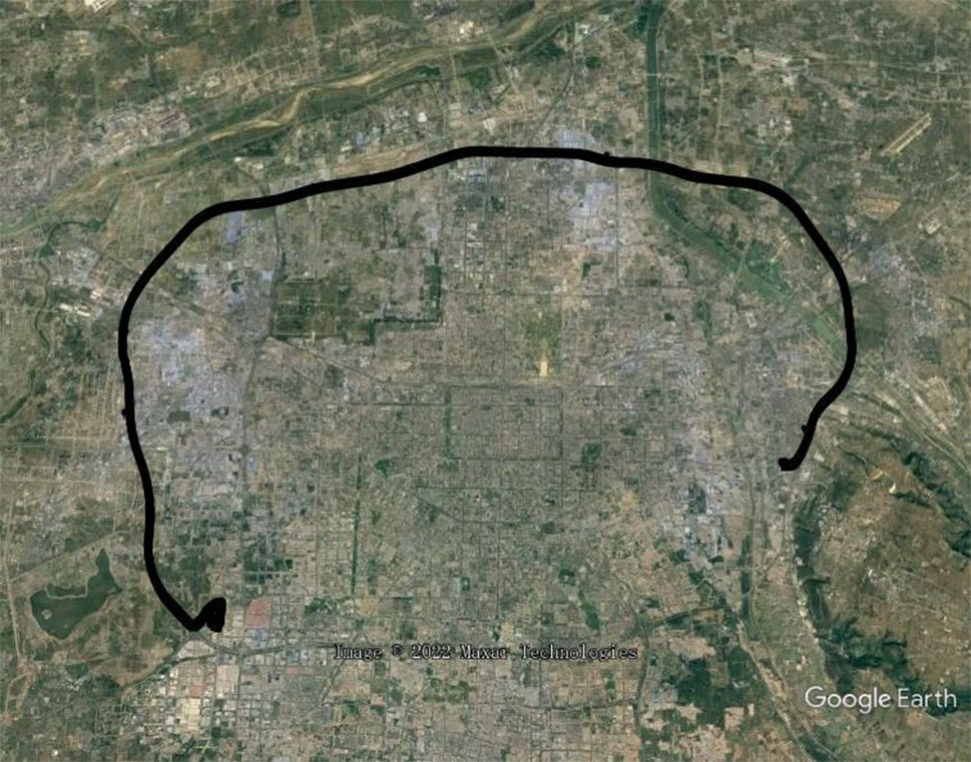
Test track of vehicle in Xi'an ring expressway.

**Figure 6:**
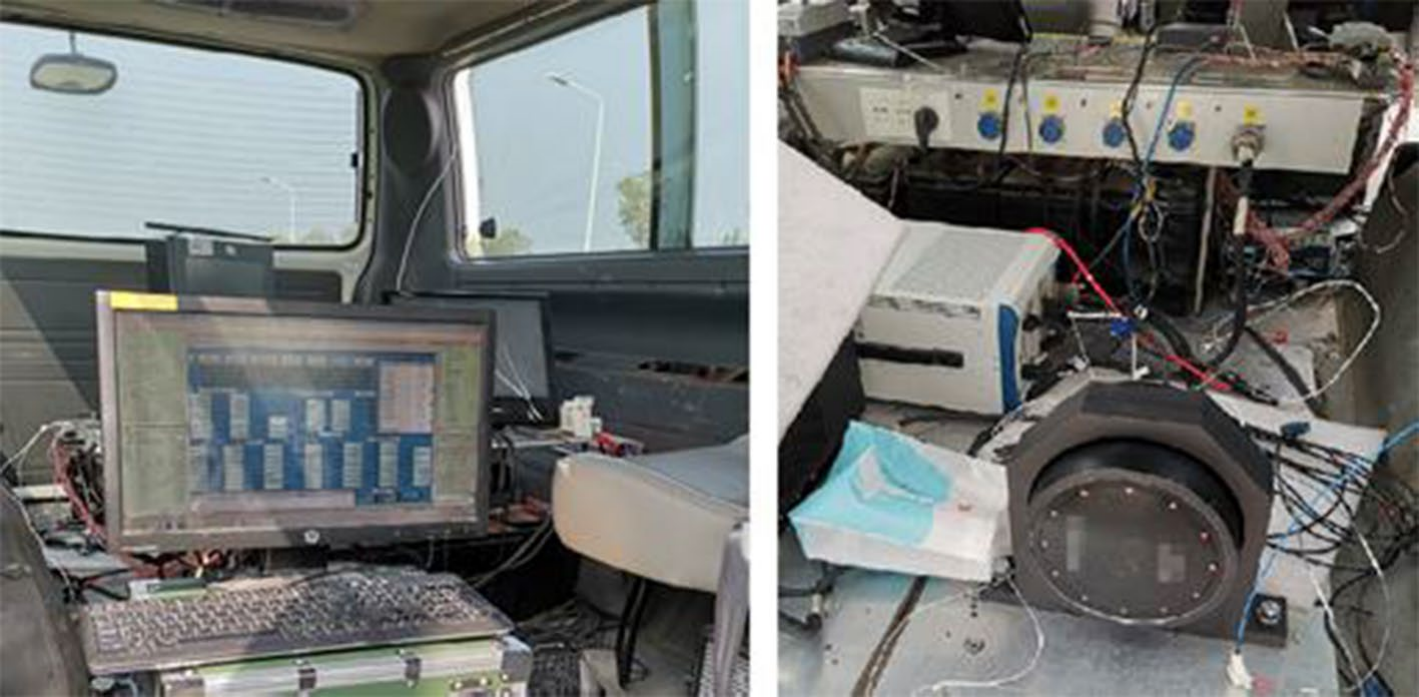
SINS/BDS integrated navigation system and test equipment.

**Figure 7:**
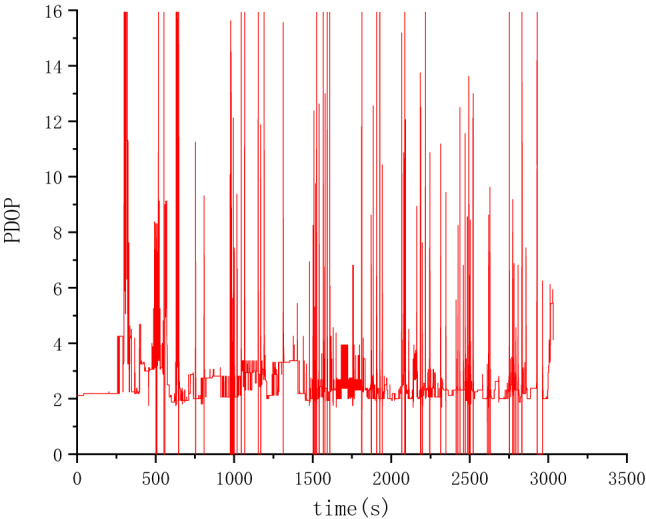
PDOP values of BDS.

A vehicle field test was carried out to verify the SINS/BDS TCINS algorithm. The test was conducted in Xi'an ring expressway, and the track is shown in Fig. 5 which is generated by Google Earth using test data. The initial latitude and longitude of the vehicle were 34.19785° and 108.82846°, respectively, the initial height was 365.1 m, and the initial velocity was 0 m/s. A high-precision integrated navigation system was equipped as the reference, called the master INS. The SINS/BDS used in this paper is called the slave INS as shown in Fig. 6. The BDS satellite number is 1–35, the Positioning Dilution of Precision (PDOP) value during the test is as shown in Fig. 7. The positioning accuracy of the satellite receiver is 10 m ($1\sigma$), the speed accuracy is 0.3 m/s ($1\sigma$), and the output frequency is 200 ms.

#### Comparison of tight coupling and loose coupling

**Figure 8:**
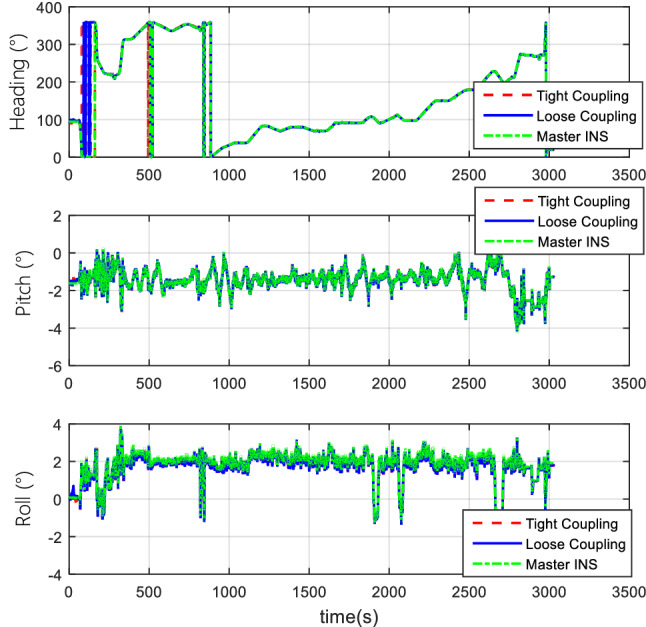
Attitude comparison of tight and loose coupling.

**Figure 9:**
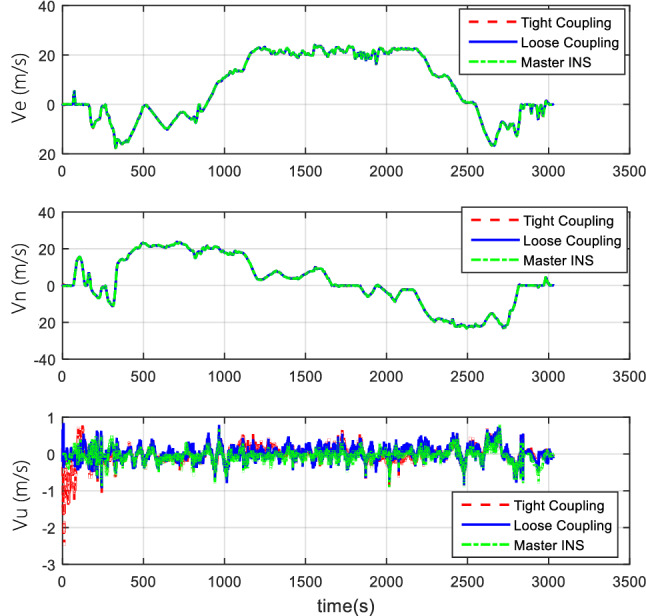
Velocity comparison of tight and loose coupling.

**Figure 10:**
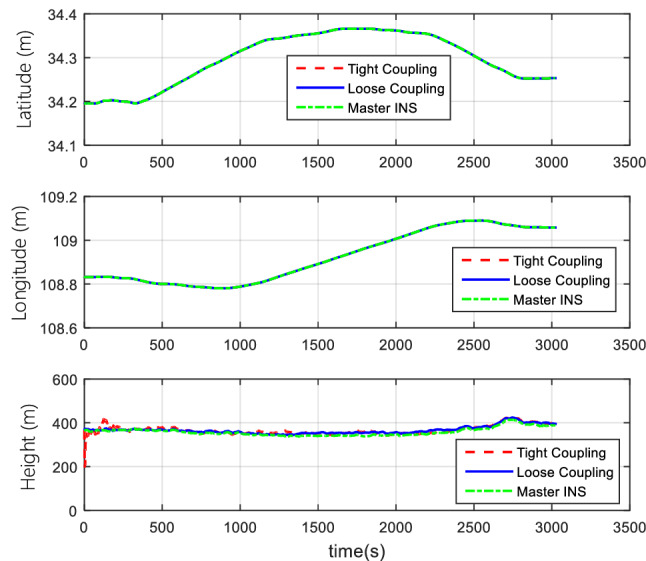
Position comparison of tight and loose coupling.

After the collection of the angular increment, velocity increment, and BDS receiver data through vehicle field tests, two combinations of tight coupling and loose coupling are adopted. The comparison results are shown in Figs. 8, 9, and 10.

In the vehicle field test, the results indicate that about the TCINS algorithm, the heading angle error is about 0.2°, the pitch angle error is about 0.02°, the roll angle error is approximately 0.03°, velocity errors in the directions of East-North-Up (ENU) are less than 0.1 m/s, and the position error is less than 10 m. On the other hand, the loosely-coupled integrated navigation algorithm has the heading angle error of about 0.3°, the pitch angle error of approximately 0.02°, the roll angle error of approximately 0.04°, the velocity errors in the directions of ENU less than 0.15 m/s, and the position error less than 15 m.

#### Comparison of the normal number of satellites and fewer-than-4 satellites

**Figure 11:**
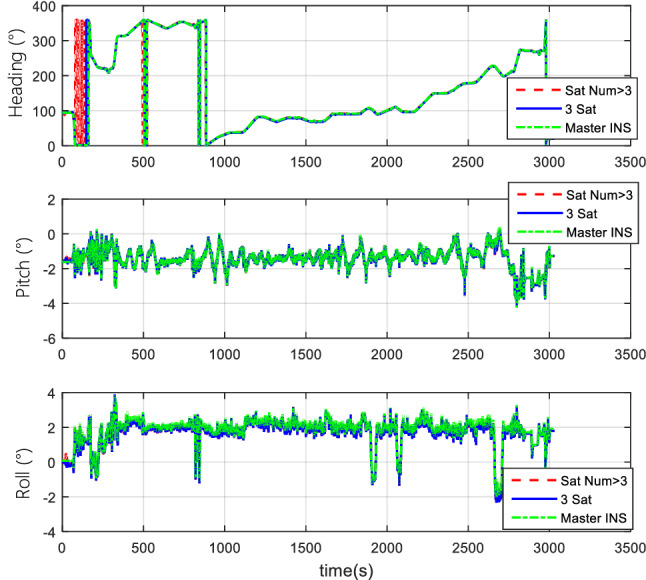
Attitude comparison of navigation by normal number of satellites and 3 satellites.

**Figure 12:**
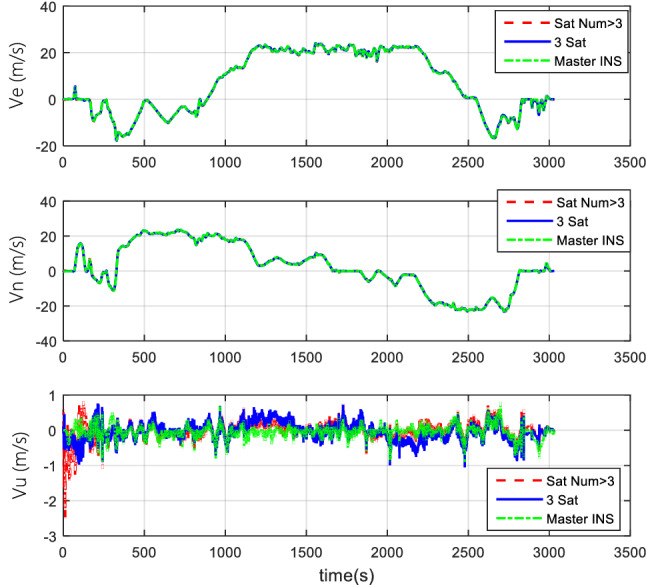
Velocity comparison of navigation by normal number of satellites and 3 satellites.

**Figure 13:**
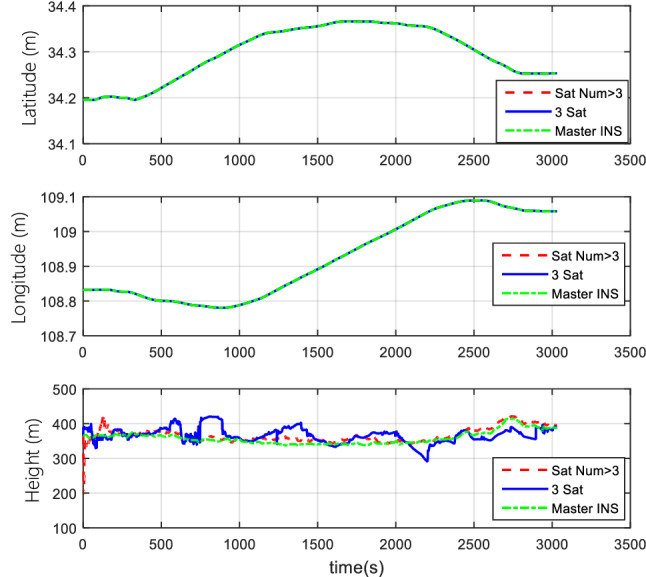
Position comparison of navigation by normal number of satellites and 3 satellites.

The vehicle field test adopts two cases of normal numbers of satellites and randomly selected three effective satellites to perform TCINS algorithms, respectively. Tight coupling can still perform integrated navigation when the number of effective satellites is fewer than 4. The comparison of simulation results between the normal number of satellites and the 3 effective satellites are shown in Figs. 11, 12, and 13.

Results demonstrate that, when only 3 effective satellites are used, the maximum heading angle error is approximately 0.5°, the pitch angle error is approximately 0.1°, and the roll angle error is approximately 0.25°. In the case of 3 effective satellites, although the navigation accuracy decreases, it can still perform tightly coupled integrated navigation normally.

### SINS/BDS integrated navigation test by HWIL simulation

**Figure 14:**
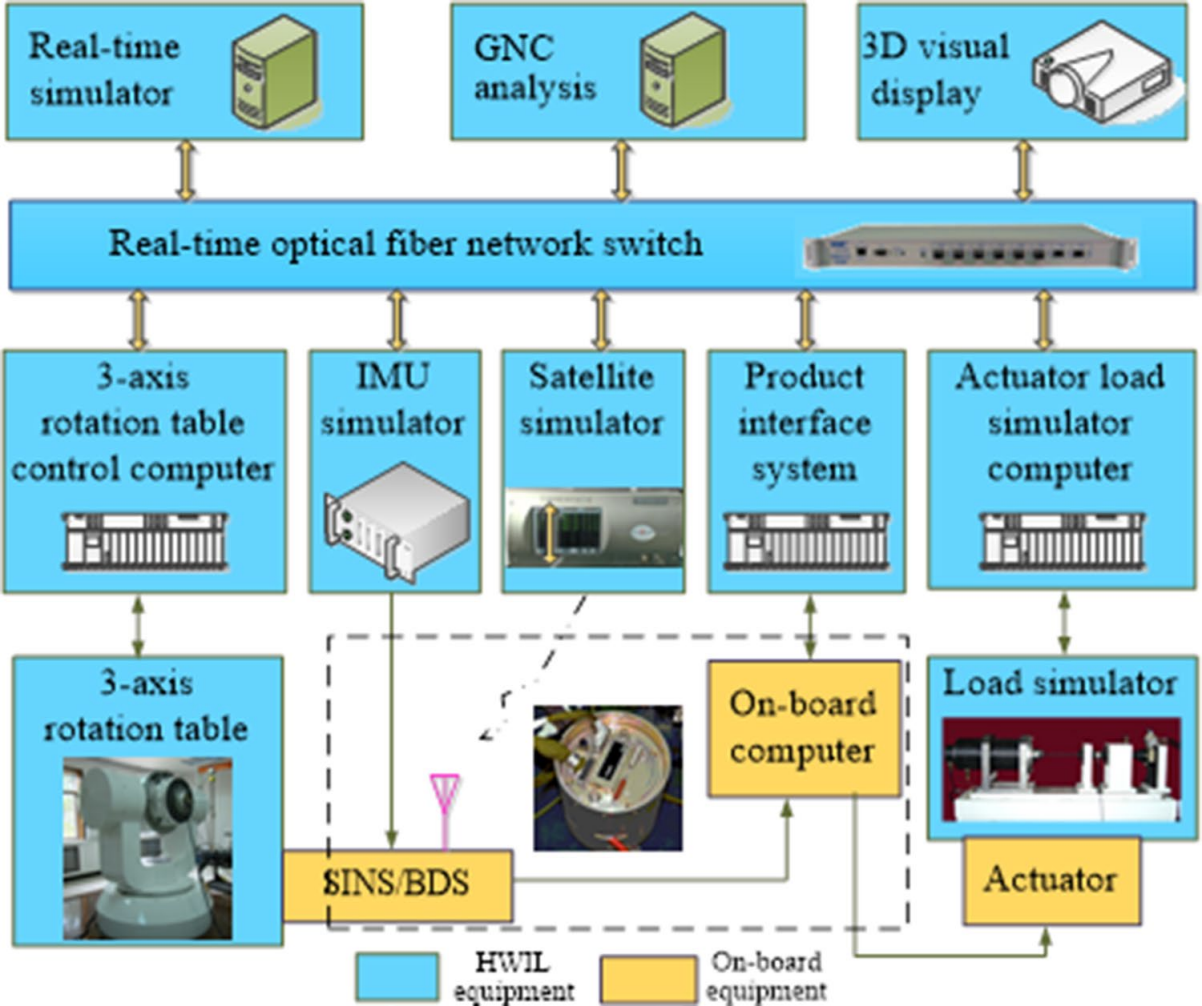
Diagram of HWIL simulation system for hypersonic vehicle SINS/BDS.

Due to the velocity limitation of field vehicle, the performance of SINS/BDS integrated navigation was verified for hypersonic velocity by the HWIL simulation, as shown in Fig. 14. The HWIL simulation tests can truly simulate the flight dynamic environment of hypersonic vehicle, and the navigation algorithm verified by the HWIL simulation tests can meet the flight requirements of hypersonic vehicle. Chen et al.^7^ has verified the effectiveness of this method. The HWIL simulation equipment includes the real-time simulator, the Guidance, Navigation and Control (GNC) analysis computer, the 3D display, the three-axis rotation table, the IMU simulator, the BDS simulator, the product interface system, and the actuator load simulator. The on-board equipment includes the on-board computer, the SINS/BDS system, and the actuator system^7^.

#### Comparison of tight coupling and loose coupling

**Figure 15:**
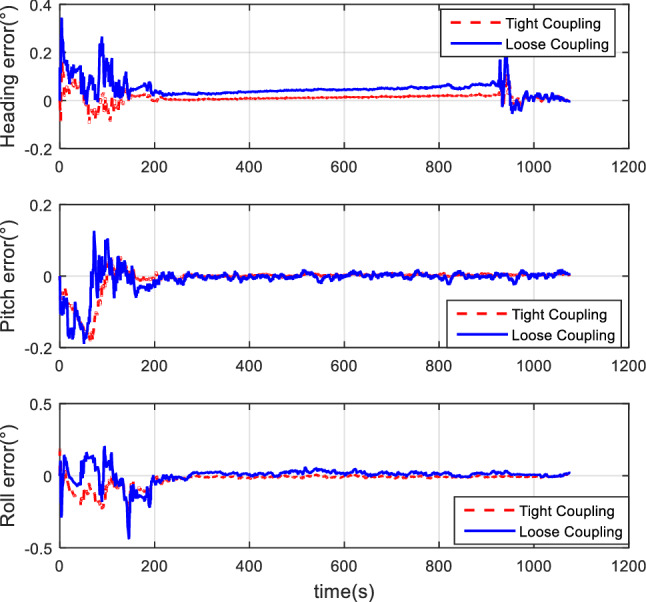
Attitude error comparison of loose and tight coupling.

**Figure 16:**
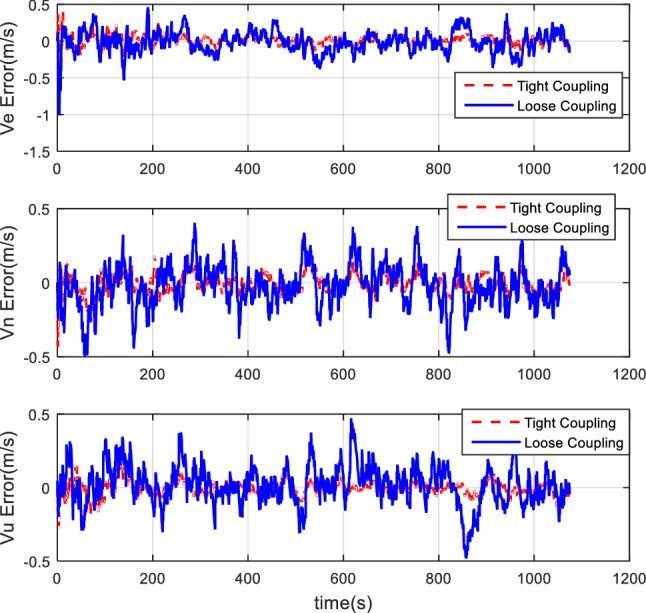
Velocity error comparison of tight and loose coupling.

**Figure 17:**
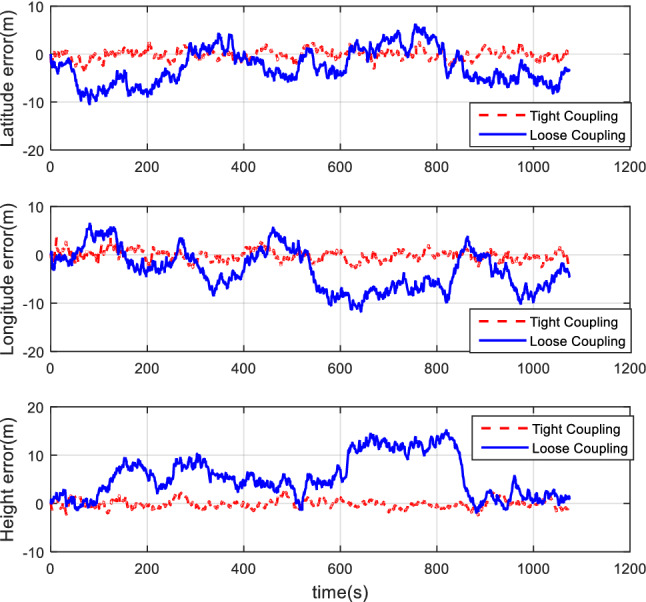
Position error comparison of tight and loose coupling.

For the same simulation conditions, the HWIL simulation adopts two combinations of tight coupling and loose coupling, respectively. The results are shown in Figs. 15, 16, and 17.

The results of the HWIL simulation test indicate that about the TCINS algorithm, errors of the heading angle, the pitch angle, and the roll angle are approximately 0.1°, 0.025°, and 0.03°, respectively. The velocity errors in the direction of ENU are about 0.3 m/s, 0.3 m/s, and 0.2 m/s, respectively. The position error is basically within 8 m. On the other hand, in the loosely-coupled integrated navigation algorithm, errors of the heading angle, the pitch angle, and the roll angle are about 0.15°, 0.03°, and 0.03°, respectively. The velocity errors in the direction of ENU are about 0.5 m/s, 0.5 m/s, and 0.32 m/s, respectively. The position error is basically within 15 m.

#### Comparison of the normal number of satellites and fewer-than-4 satellites

**Figure 18:**
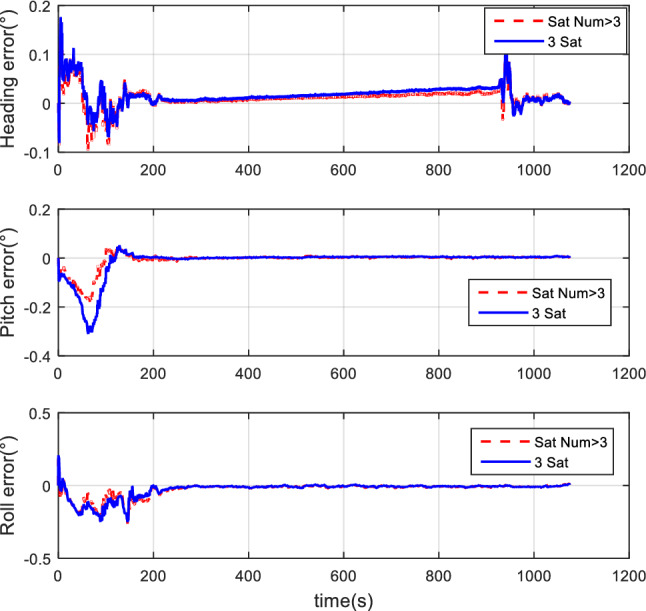
Attitude comparison of navigation by normal number of satellites and 3 satellites.

**Figure 19:**
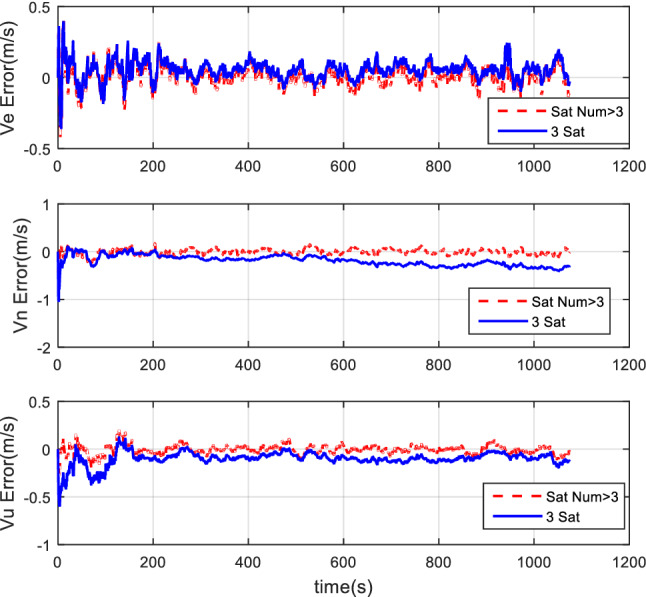
Velocity comparison of navigation by normal number of satellites and 3 satellites.

**Figure 20:**
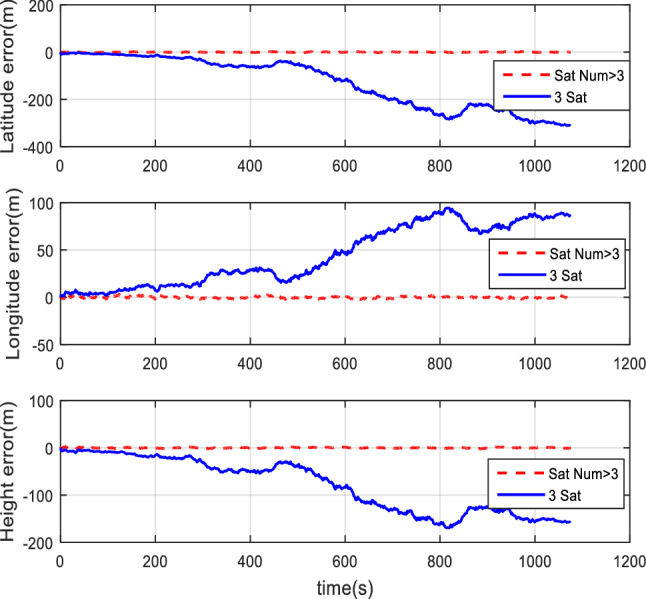
Position comparison of navigation by normal number of satellites and 3 satellites.

The HWIL simulation still adopts two cases of normal numbers of satellites and randomly selected three effective satellites to perform TCINS simulations, respectively. The HWIL simulation results are verified that the tight coupling can still perform integrated navigation normally when there are fewer than 4 satellites. The comparison of simulation results between the normal number of satellites and the 3 effective satellites are shown in Figs. 18, 19, and 20.

When 3 effective satellites are adopted, the heading angle error is about 0.2°, the pitch angle error is approximately 0.03°, and the roll angle error is approximately 0.04°. The velocity errors in the directions of ENU are about 0.3 m/s, 0.6 m/s, and 0.8 m/s, respectively. The maximum horizontal position error and the height error are 180 m and 200 m, respectively.

## Conclusions

In this study, we designed a TCINS algorithm based on the architecture of SINS/BDS integrated navigation system of hypersonic vehicle. Owing to the normal gravity model is no longer applicable in near space, the algorithm uses a J2 gravity model, which is appropriate for near space flight heights over 20 km. We designed the numerical update algorithm of inertial navigation for hypersonic vehicle, and gave the scheme to judge the validity of BDS satellite data. The proposed navigation algorithm was verified by vehicle field tests; hardware-in-the-loop simulation tests are also conducted in order to truly simulate the dynamic environment of hypersonic vehicle flight. Although satellite navigation is susceptible to interferences, the tightly coupled applications can overcome the problem of navigation from fewer than 4 satellites.
